# Treatment Planning for Self-Shielded Radiosurgery

**DOI:** 10.7759/cureus.1663

**Published:** 2017-09-08

**Authors:** John R. Adler, Achim Schweikard, Younes Achkire, Oliver Blanck, Mohan Bodduluri, Lijun Ma, Hui Zhang

**Affiliations:** 1 Department of Neurosurgery, Stanford University School of Medicine; 2 Institute for Robotics and Cognitive Systems, University of Luebeck, Institute for Robotics and Cognitive Systems, University of Lubeck; 3 Zap Surgical Inc.; 4 Department for Radiation Oncology, University Medical Center Schleswig-Holstein, Campus Kiel, Germany; 5 Department of Radiation Oncology, University of California, San Francisco

**Keywords:** medical robotics, treatment planning, robotic radiosurgery

## Abstract

A five degree of freedom, robotic, radiosurgical system dedicated to the brain is currently under development. In the proposed design, the machine is entirely self-shielded. The main advantage of a self-shielded system is the simplification of the system's installation, which can reduce the cost of radiosurgery. In this way, more patients can benefit from this minimally invasive and highly effective type of procedure. For technical reasons, space inside the shielded region is limited, which leads to constraints on the design. Here, two axes of rotation move a 3-megavolt linear accelerator around the patient’s head at a source axis distance of 400 millimeters (mm), while the integrated patient table is characterized by two additional rotational, and one translational, degrees of freedom. Eight cone collimators of different diameters are available. The system can change the collimator automatically during treatment, using a collimator wheel. Since the linear accelerator can only move with two rotational axes, it is not possible to reposition the beam translationally (as it is in six degrees of freedom robotic radiosurgery). To achieve translational repositioning, it is necessary to move the patient couch. Thus, translational repositioning must be kept to a minimum during treatment. Our goal in this contribution is a preliminary investigation of dose distributions attainable with this type of design. Thus, we do not intend to design and evaluate the treatment planning system itself, but rather to establish that appropriate dose distributions can be achieved with this design under realistic clinical circumstances. Our simulation suggests that dose gradients and conformity for complex target shapes, corresponding to state-of-the-art systems, can be achieved with this construction, although a detailed evaluation of the system itself would be needed in the future.

## Introduction

In radiosurgery, beams from many different directions deliver a focused dose of radiation to a targeted lesion/tumor. Two basic technical platforms for radiosurgery can be distinguished:

- Gamma Knife® radiosurgery (Elekta, Inc., Stockholm, Sweden) 

- linear accelerator (LINAC) radiosurgery

The Gamma Knife is a radiosurgical system, in which radiation beams from 192 directions converge at a single point in space: an isocenter [1]. The beams are produced by 192 cobalt-60 sources. During treatment, the patient is immobilized via a stereotactic frame and placed such that the target is located at the intersection point of the beam axes. The operator can choose between three different (cylindrical) beam diameters (4, 8, or 16 mm). To select a specific diameter, the operator attaches a helmet-type structure to the patient’s head. The helmet is an assembly of metal collimators for the beams.

By contrast, LINAC radiosurgery works with a single beam source. A motor-driven gantry, or robot, moves the source to produce beams from different directions. An example for a LINAC-type radiosurgery system is the CyberKnife® (Accuray, Inc., Sunnyvale, CA, USA) [2]. Typically, a cylindrical collimator or a multileaf collimator is mounted in front of the beam source. In the case of a cylindrical collimator, the beam has a cylindrical shape [3-4]. Multileaf collimators have been designed to improve the conformity of the dose distribution, especially for non-spherical targets, and to reduce treatment time. However, multileaf collimators are costly and do not always produce optimal results [5]. Given the space limitations of the proposed design, cone collimators have substantial benefits.

Although a five degree of freedom (5-DoF) robotic radiosurgical system dedicated to the brain was first proposed in 1991 [6], the implementation of this approach has only recently started. In the proposed design, two axes of rotation move a linear accelerator around the patient’s head, while the integrated patient table is characterized by two additional rotational, and one translational, degrees of freedom. The two rotational table axes generate approximations for translational motion of the patient's head.

As noted, the beam is moved by two rotational axes. Thus, we cannot reposition the beam translationally, as in the CyberKnife system. To obtain a new translational position of the beam with respect to the patient, we must move the patient couch. Therefore, the treatment planning system should limit translational repositioning of the beam to a minimum. The wheel can be rotated by a motor and carries eight cylindrical collimators of different diameters. Thus, we can rapidly switch the collimator many times during treatment without user interaction and without treatment interruption.

In the present study, we investigate the dose distributions attainable with this type of kinematics. Given the trade-offs between treatment time, planning time, and distribution characteristics, our goal is to evaluate the kinematic construction of the system and the collimator range with diameters of 4.0, 5.0, 7.5, 10.0, 12.5, 15.0, 20.0, and 25.0 mm, for the short-source axis distance of 400 mm. Notice that it is not our goal here to design, nor to evaluate the treatment planning system itself, but to establish that the proposed design can generate distributions suitable for routine clinical practice. Nonetheless, to reach this goal, we must derive and implement a suitable treatment planning method.

As a basis of treatment planning methods for the Gamma Knife, Ferris, et. al. [7-8] derived a sphere packing algorithm based on mixed-integer programming. The main advantage of mixed-integer programming is that the exact number of spheres to be allocated can be specified in advance by the user. However, mixed-integer programming is far from practical for this application, especially since it is almost always necessary to search the parameter space via repeated calls to the system. For example, even an experienced user would have difficulty to estimate the number of spheres to allocate in advance, given a tumor's shape. Furthermore, from an application point of view, the advantage of being able to determine the exact number of spheres in advance is very small. The interaction of sphere packing and computing individual beam weights turns out to be very useful. Notice that the Gamma Knife cannot allocate individual weights to beams [1]. We can hereby show that the motion flexibility of a dedicated five degrees of freedom radiosurgical robot allows for producing highly conformal dose distributions, comparable to the results achievable with multileaf collimators and/or very sharp dose gradients.

## Materials and methods

Given mechanical limitations of the system, we can produce beams from a range of directions in space of an area slightly more than 2 $\pi$, where beam axes all intersect at a single point. Thus, the treated volume is a sphere. However, we cannot only move the gantry but also the couch. Thus, we can reposition the patient by moving the patient couch.

**Figure 1:**
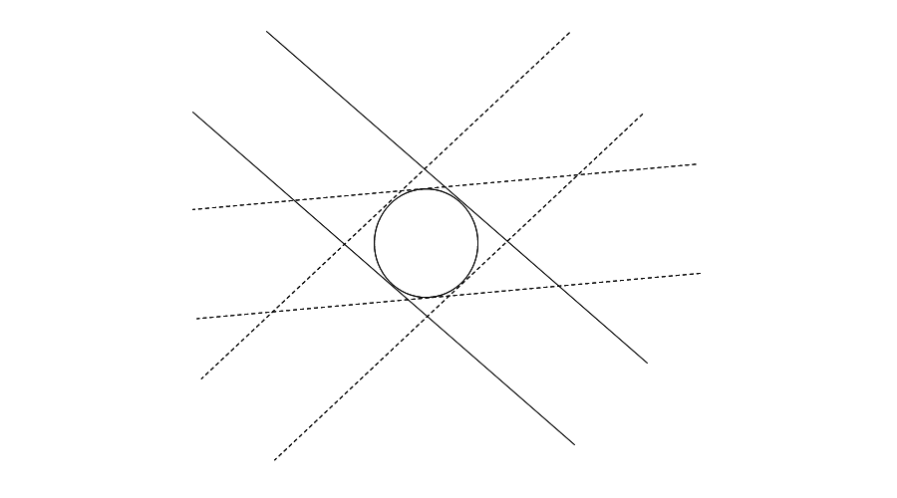
Single isocenter treatment Irradiating a target from many directions in space with a cylindrical beam produces a treated volume of spherical shape.

**Figure 2:**
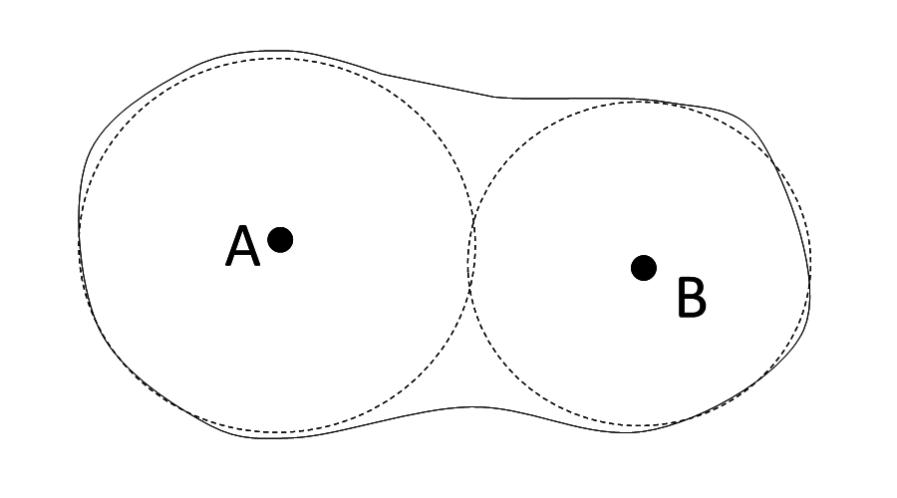
Two spheres covering non-spherical target volume For targets with a non-spherical shape, we place several spheres into the target. Each of these spheres is then treated as in Figure 1.

We illustrate the treatment process in Figures 1-2. Figure 1 shows a tumor with a spherical shape. We position the patient in such a way that the axes of the beams (produced by different beam angles) intersect at the center of the tumor. Then, the treated volume will be a sphere. Figure 2 shows a non-spherical tumor. Here, we proceed in much the same way, producing first a spherical treated volume by positioning the patient such that the beam axes cross at a single point. Subsequently, we reposition the patient to point B and produce a second sphere. This procedure is similar to the Gamma Knife treatment protocol.

 

Sphere packing

To plan a treatment, we must first place spheres as in Figure 2. This can be difficult and time-consuming, especially for complex tumor shapes. We have chosen to implement an automatic sphere-packing scheme to support this process. The methods for this automatic scheme will be described next.

We place the spheres such that they do not intersect one another, and also do not intersect healthy tissue surrounding the tumor. Since there are eight collimator sizes, we can place spheres of eight different sizes, matching the sizes of the collimators (4.0 mm, …, 25.0 mm). It would also seem possible to place the spheres such that intersections between spheres are allowed. However, we will not use this possibility, for reasons to be discussed below. The algorithm places the spheres one-by-one, into the target, starting with the largest size. Our primary planning goal is conformity, i.e the treated volume should match the target shape. To this end, it is useful to place the spheres in such a way that they cover as many surface voxels as possible. Thus, for example, let the first sphere we can fit inside the tumor be a sphere of size 25.0 mm. Then, there may be several possible distinct placements for this first sphere. Among the available placements, we choose the placement covering the largest number of surface voxels. This is done to avoid fragmentation of the available space. An additional rationale for this heuristic method stems from the fact that the surface carries the most information on the shape of the target.

**Figure 3:**
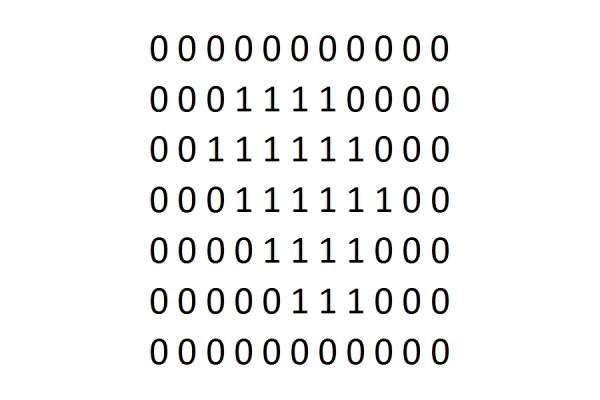
Binary grid Binary grid for representing tumor volume. A 3D representation uses a series of such cross-sections.

**Figure 4:**
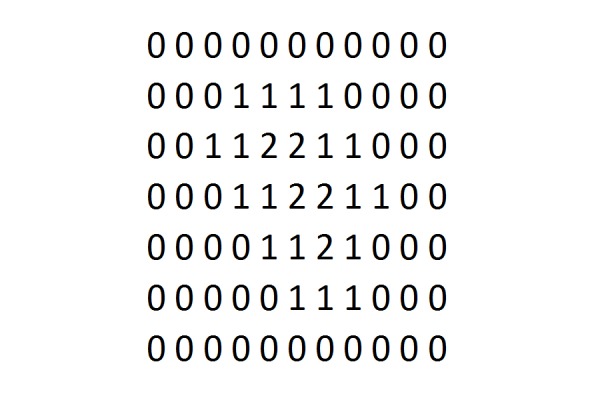
Morphological grid

**Figure 5:**
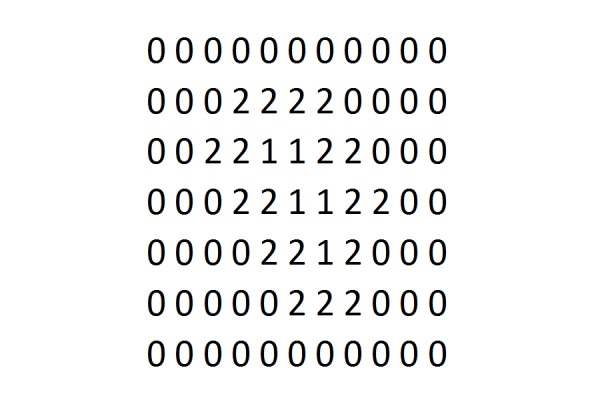
Reverse morphological grid

When applying this sphere packing method, how do we find the sphere closest to the surface? We compute what we call a reverse-morphology grid for the target volume. This process is illustrated in Figures 3, 4, and 5. We place a dense grid of points over the anatomic region of interest, containing the target tumor in its center. Typically, this grid is a point grid of dimension 64 x 64 x 64 or 128 x 128 x 128 voxels, where each voxel represents a volume of fixed size, i.e. one cubic millimeter or less. Positive voxels (i.e., tumor voxels) are labeled by a 1 (Figure 3). Surrounding soft tissue is represented by a zero. Thus, the grid in Figure 3 is a binary grid. A morphological grid labels the voxels according to their distance from the surface, as shown in Figure 4. Now, a reverse morphology grid simply reverses this distance, i.e., voxel values inside the target are counted from the highest to the lowest value, instead of from lowest to highest, such that surface voxels have the highest values and center voxels have the lowest value (Figure 5). Notice that Figures 3, 4, and 5 are two-dimensional illustrations of grids. The grids used here are three-dimensional (3D) grids.

 

Having computed a reverse morphology grid, it is easy to select the candidate sphere center. We assign a score to each candidate sphere. The score is simply the sum of all labels of voxels covered by the sphere. Amongst the candidate spheres, we choose the sphere with the highest score. Any remaining ambiguities are resolved by choosing a center at random.

If no more spheres of this current (largest) size will fit, we repeat the same process for the next smaller sphere, until all sizes have been used. In rare cases, the tumor is so small that no sphere, not even of the smallest size (i.e., 4.0 mm) will fit. In such cases, we slightly enlarge the tumor, by adding a tight margin around it, until a sphere will fit.

In practice, it is necessary to be able to reduce the number of spheres thus placed. As noted above, reducing the number of spheres will directly reduce the number of couch repositioning motions. Hence, we can thereby reduce the number of images taken during treatment, and thus reduce treatment time. Translational repositioning can only be done by moving the couch. This, in turn, will require additional imaging and increase treatment time. To reduce the number of spheres, we introduce a new parameter controlling the allocation of the spheres. This parameter is the spacing between spheres. Thus, after having placed the first sphere, we not only require that the next sphere must not intersect any spheres already placed, but in addition, we require that it remains at a fixed distance (specified by this input parameter) from all spheres already placed. The same is done for all subsequent placements.

Notice that our beam placement strategy outlined so far is a heuristic strategy. Having placed all spheres, we put a fixed number of beams through all spheres from random angles. The number of beams per sphere is an input parameter. For typical treatments, up to 10 or more spheres are packed inside the target. We then allocate a fixed number of beam directions to each of the spheres. Typically, 60-100 beams are allocated per sphere. Notice that all beam axes intersect the center of at least one sphere, i.e., if b is one such beam allocated to a sphere-s, then the axis of b will cross the center of s. Notice also that all beams crossing the midpoint of a given sphere have the same diameter, namely the diameter of the sphere itself.

Beam weights

The treatment beam is a 3-megavolt photon beam. We activate the beam at different configurations in space. The duration of activation corresponds to the dose delivered by each beam direction. This duration will be referred to as the weight of the beam direction.

The possibility to compute an individual weight for each beam direction (or each beam, for short) turns out to be of utmost importance towards the goal of conformity.

Suppose we have placed $n$ beams, in the preceding step, i.e., the sphere packing step. Let $p$ be a point in the anatomical region of interest. Then $p$ will be contained not in all, but in a subset of the $n$ beams; (notice that $p$ will only be inside of, at most, one sphere, but it can nonetheless be contained in more than one beam!). Let $b_1,&hellip;,b_s$ be the subset of beams containing $p$. To ensure that $p$ will absorb a fixed dose $d$, the sum of all doses to the beams $b_1,&hellip;,b_s$ must be equal to d. Now let $x_i$ be the duration of activation of beam $b_i$. This duration of activation corresponds to a dose delivered by this beam. To simplify the discussion, we identify this dose value and the duration of activation. Then, we have the equation:

(1)        $x_1 + x_2 + &hellip; + x_s = d$

In practice, we cannot achieve dose distributions where each point receives exactly a prescribed dose $d$. Rather, we must set thresholds, i.e., we must ensure that upper and lower bounds on the dose to each point in the region of interest will be met.

Thus, we replace the single equation in (1) by the following two inequalities:

(2)       $x_1 + x_2 + &hellip; + x_s \leq u$

and

(3)       $x_1 + x_2 + &hellip; + x_s \geq l$

This means that the dose at $p$ should be larger than or equal to a lower threshold $l$, and less than an upper threshold $u$.

To begin the process of computing beam weights, we again consider a voxel grid of size 64 x 64 x 64 containing the target in its center. We set up inequalities of the form shown in (2) and (3) for each voxel. This gives rise to a set of inequalities which we can solve with linear programming.

Linear programming is a general tool from mathematical optimization. In the standard version of linear programming, we have inequalities, such as the above inequalities in (2) and (3), and an objective function. The objective function is linear, and we can express, for example, the objective of minimizing the integral dose in this function. The constraint inequalities correspond to dose thresholds in the tumor, the surrounding tissue, and critical healthy organs close to the target.

As an alternative to linear programming, we can use quadratic programming by slightly modifying the constraint formulation. This leads to a different system of inequalities.

To set up the system of inequalities and an objective function for quadratic programming, we proceed as follows. We consider a voxel, p, on the surface of the target.

Again, assume the voxel $p$ is contained in beams $b_1, &hellip; , b_s$, but in no other beams, among the beams defined in the sphere packing step. (When we speak about a beam ‚containing‘ a voxel, we mean that a geometric cylinder of the beam’s collimator diameter contains the voxel. However, the beam is not an ideal cylinder. In the discussion below, we will see how to adjust for that fact, and include penumbra effects into the calculation.) Now suppose we have prescribed a dose $d$ for the target. As above, variables $x_1 + x_2 + &hellip; + x_s$ specify the dose to the beams $b_1, &hellip; , b_s$. To achieve conformity, we would like to minimize deviations from the prescribed dose $d$, especially on the surface of the target. We measure such deviations with a least squares distance. Thus, we require that

(4)                    $(x_1 + x_2 + &hellip; + x_s - d)^2$

be minimal.

Now, if $q$ is a second surface voxel, we obtain a similar expression for $q$. Summing up these quadratic expressions for all surface voxels gives a quadratic objective function F of the dose variables $x_i$ for the beams. Thus, minimizing the function F will reduce the dose deviation at the tumor surface. We can reduce the size of the objective function by considering subsets of the surface voxels.

We can now add linear inequality constraints on the dose distribution to our quadratic program, i.e., we can include lower bounds for the dose at fixed points in the anatomy in much the same way as in equation (2), i.e. we can constrain dosage in critical organs near the target. Below, we will discuss the case of infeasible constraints. In most cases, it is useful to include a lower bound $l$ for the dose at each tumor voxel. This means, our quadratic program will only accept solutions, where the minimum dose $l$ is absorbed by each tumor voxel.

Finally, we can place a shell of voxels around the target. This means we apply the quadratic minimization not to the surface of the target, but to a shell of voxels at a fixed distance, $f$, from the target. In this way, we can minimize the dose to the soft tissue around the target. This is equivalent to optimizing the gradient of the dose. The shell distance $f$ is again an input parameter and is set by the user. In addition, we can impose local shell constraints, i.e., we can optimize the gradient in certain subregions close to the target. Such subregions can be regions close to critical healthy organs near the target. In the case of a shell, equation (4) can be simplified. Here, our goal is to minimize dose to the shell, in a least squares sense. Thus, the quadratic objective function is a sum of expressions of the form

(5)                    $(x_1 + x_2 + &hellip; + x_s)^2$                                                                          

Thus, we form a single expression consisting of subexpressions of the form (5) for all voxels on the shell (or the surface).

In summary, we obtain a quadratic program describing the desired dose distribution in such a way that conformity and dose gradients are optimized. At the same time, the prescribed target dose is specified via constraint inequalities. Notice that not all types of constraints are always needed. In many cases, lower bounds for all tumor voxels in conjunction with single a shell constraint are fully sufficient. However, the user can add a variety of other types of constraints, i.e., lower and upper bounds in conjunction with quadratic constraints.

In linear and quadratic programming, an exception can arise if the input constraints are infeasible. For example, the user could input a lower bound for a voxel, and at the same time, input an upper bound for the same voxel, where the lower bound is higher than the upper bound. The user must then modify the thresholds accordingly. Linear and quadratic programming can detect this condition and report it to the user. We have found that a very simple way to avoid such conditions is to only allow for lower bounds at voxels and impose a shell constraint as in (5), in conjunction with quadratic programming. The shell constraint can be placed tighter in the vicinity of organs at risk.

Dosimetric beam model and penumbra

For a prototype system (3-megavolt photon beam with 400 mm source-axis distance) measurements for off center ratios (OCR) tables, tissue-phantom ratios (TPR) table, and output factor (OF) tables were recorded.

Table 1 lists penumbra widths (80%-20%) taken from these measurements for a depth of 50 mm:

 

**Table 1:** Penumbra Widths mm: millimeter

Collimator (mm)	4	5	7.5	10	12.5	15	20	25
Penumbra Widths (80-20, mm)	1.8	2.0	2.0	2.2	3.0	3.4	3.4	4.1

We can now directly include these measurements (TPR, OCR, OF) into inverse planning by adding appropriate coefficients to our linear or quadratic program. Thus, linear and quadratic programming support constraints of the form

(6)                        $a_1 x_1 + a_2 x_2 + &hellip; + a_s x_s \geq l$

 

Here, the constant coefficients $a_1i$ represent the dose per beam and voxel, obtained as a factor, from forward dosimetry. For example, the coefficient $a_1$ is the dose factor for the first constraint voxel stemming from beam 1. We compute this factor using the measured tables, in the same way as in standard forward dosimetry.

## Results

**Figure 6:**
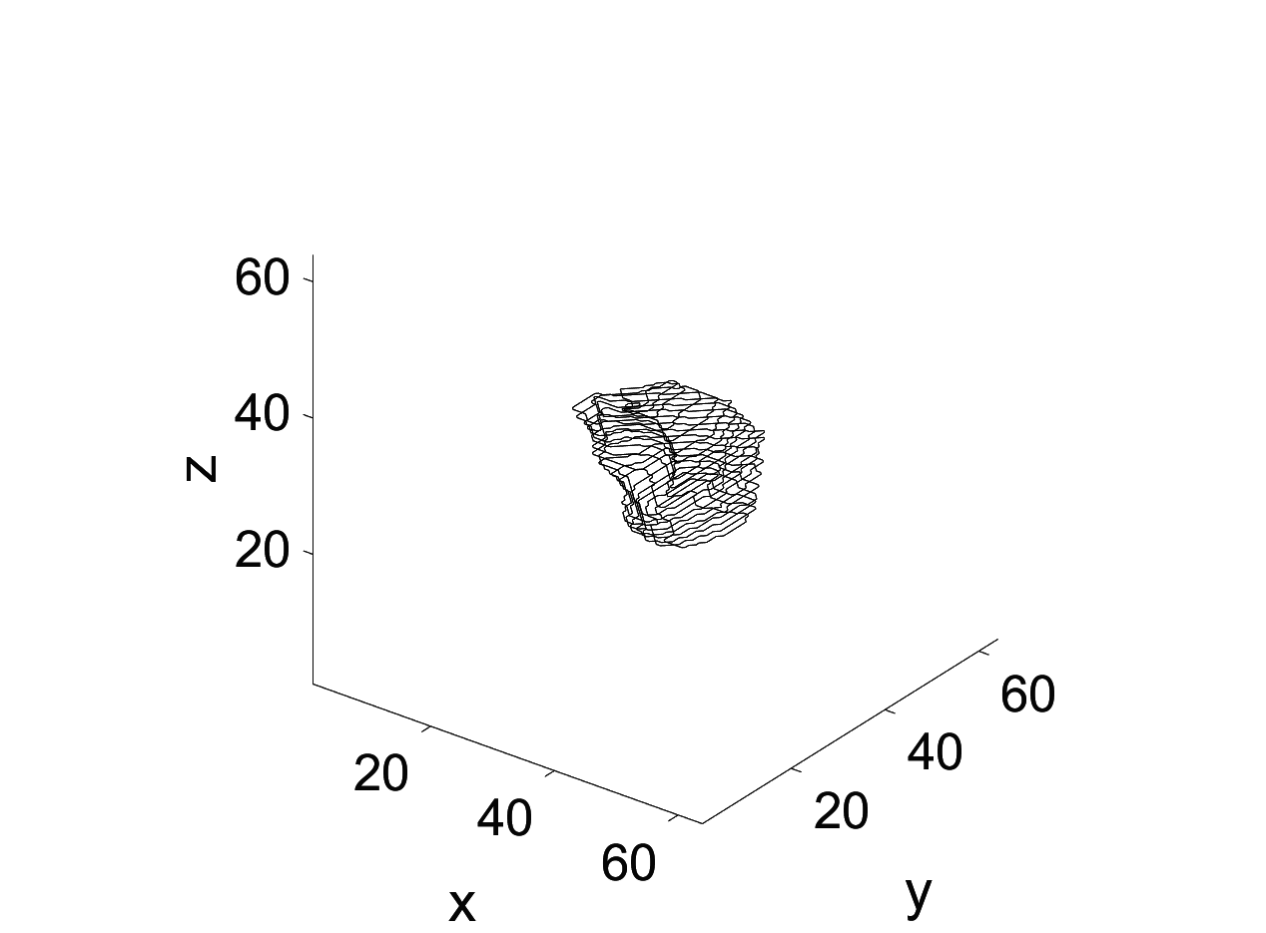
Tumor contours

In an evaluation, we considered three typical clinical tumor cases collected from several institutions. In all cases, we applied our sphere packing in conjunction with simple quadratic programming. The thresholds were such that we set a lower bound of 2,000 centigray (cGy) for all target voxels and simple QP-objective function constraints for all shell voxels. This amounts to minimizing the squared sum of dosage to all shell voxels as described in the previous section. Notice that problems with infeasibility cannot arise under these constraints, as long as we make sure that each tumor voxel is covered by at least one beam. We can easily check this in the beam placement algorithm and add beams through an uncovered voxel if needed. The contours for the first case (Case 1) are shown in Figure 6. The figure shows the tumor contours delineated in all slices of a tomographic image data set.

**Figure 7:**
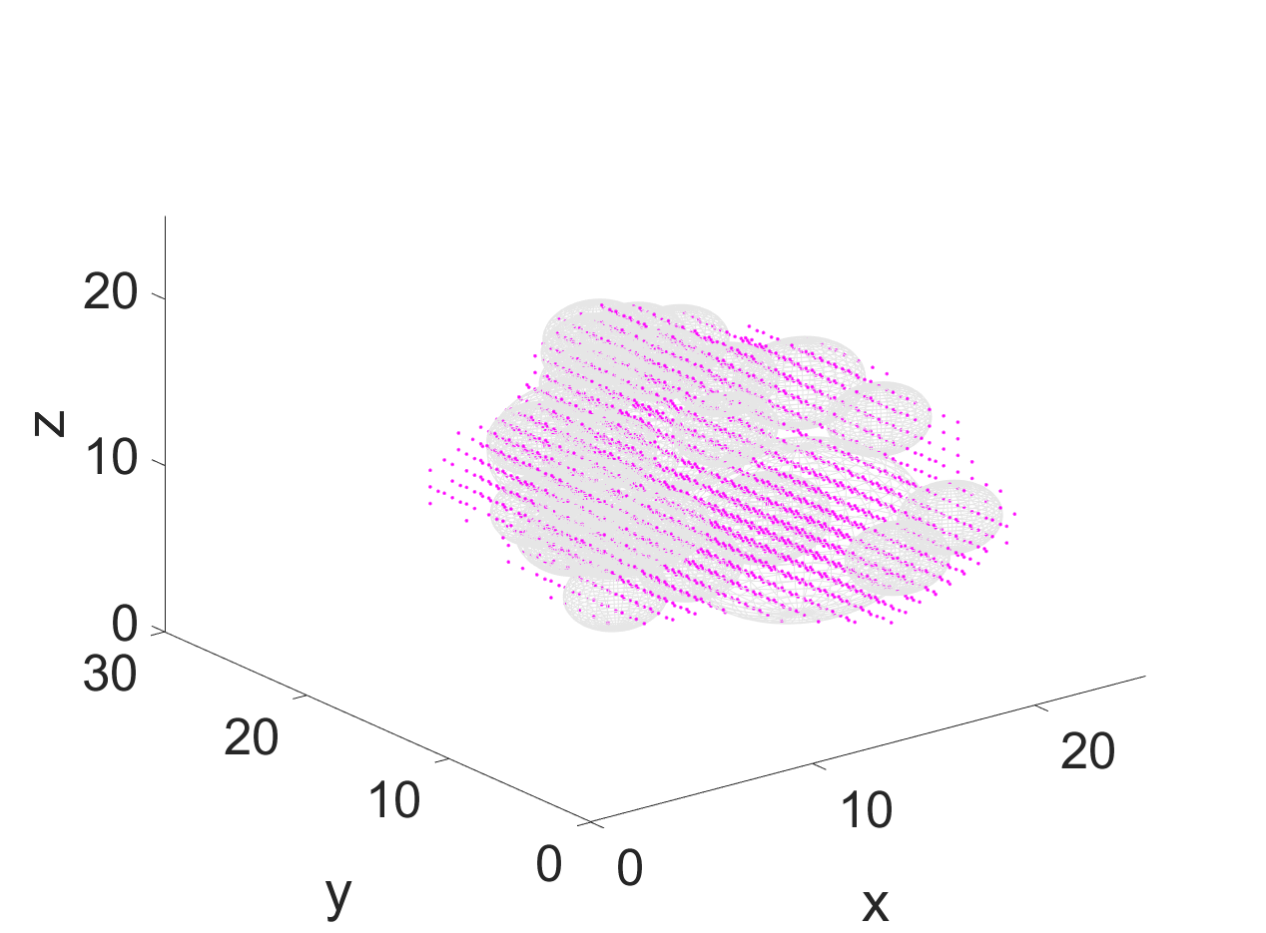
Result of sphere packing

We begin by placing the spheres. Starting with a spacing parameter of zero between the spheres, the algorithm described above returns 27 spheres, as shown in Figure 7. The points shown in magenta are the tumor voxels. Notice that our sphere packer places spheres inside the target so that some voxels are not covered by spheres. However, these voxels can still be covered by the beams defined by these spheres.

**Figure 8:**
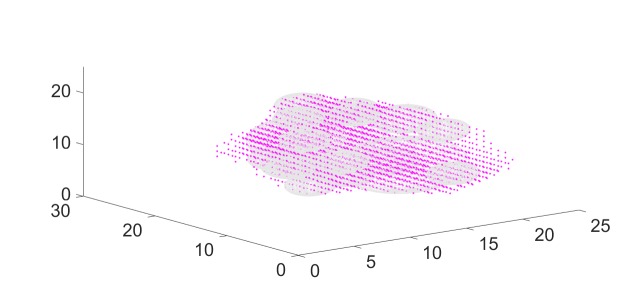
Reducing the number of spheres by adding a spacing between spheres

We can reduce this number of spheres by prescribing a non-zero spacing here of 0.3 mm. In this case, the sphere packing algorithm allocates only 19 spheres, as shown in Figure 8. As noted above, all beams pass through the centroid of at least one sphere.

**Figure 9:**
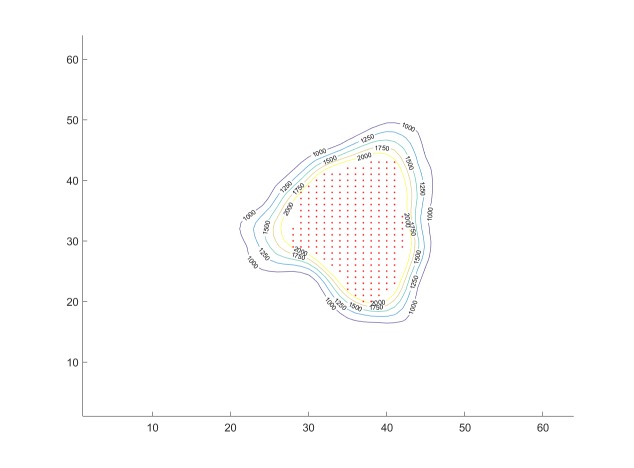
Isodose curves for an axial slice in Case 1. Red dots: tumor voxels

We next place 140 beams through each of the 19 spheres, resulting in a total of 2,660 candidate beams for beam weight computation with quadratic programming. We set thresholds as described above and compute the beam weights with quadratic programming. In this case, we find that the algorithm assigns non-zero weights to 440 of the candidate beams. (The majority of the beams receive weight zero.) The result is shown in Figure 9. The figure shows one cross-sectional slice of the target. The dose distribution is visualized via isodose lines. We see that all tumor voxels (red dots) are contained in the 2,000 cGy isodose curve, so that all tumor voxels receive a dose of 2,000 cGy or higher. In this case, the conformity index (CI) [9] (for a prescribed dose of 2,000 cGy) is well-defined and we can compute it as the ratio between the tumor volume and the volume contained in the 2,000 cGy isodose region. Similarly, the steepness of the dose gradient in the region surrounding the target can be measured via a gradient index (GI) [10], defined as the ratio between the volume receiving 100% of the prescribed dose (2,000 cGy in our case) and 50% of the prescribed dose (1,000 cGy in our case). For our first sample case, we obtained the values for CI = 1.20 and for GI = 2.64. All values were obtained using precise measurements for off-center ratios, tissue-phantom-ratios, and output factors for the collimators and the LINAC.

**Figure 10:**
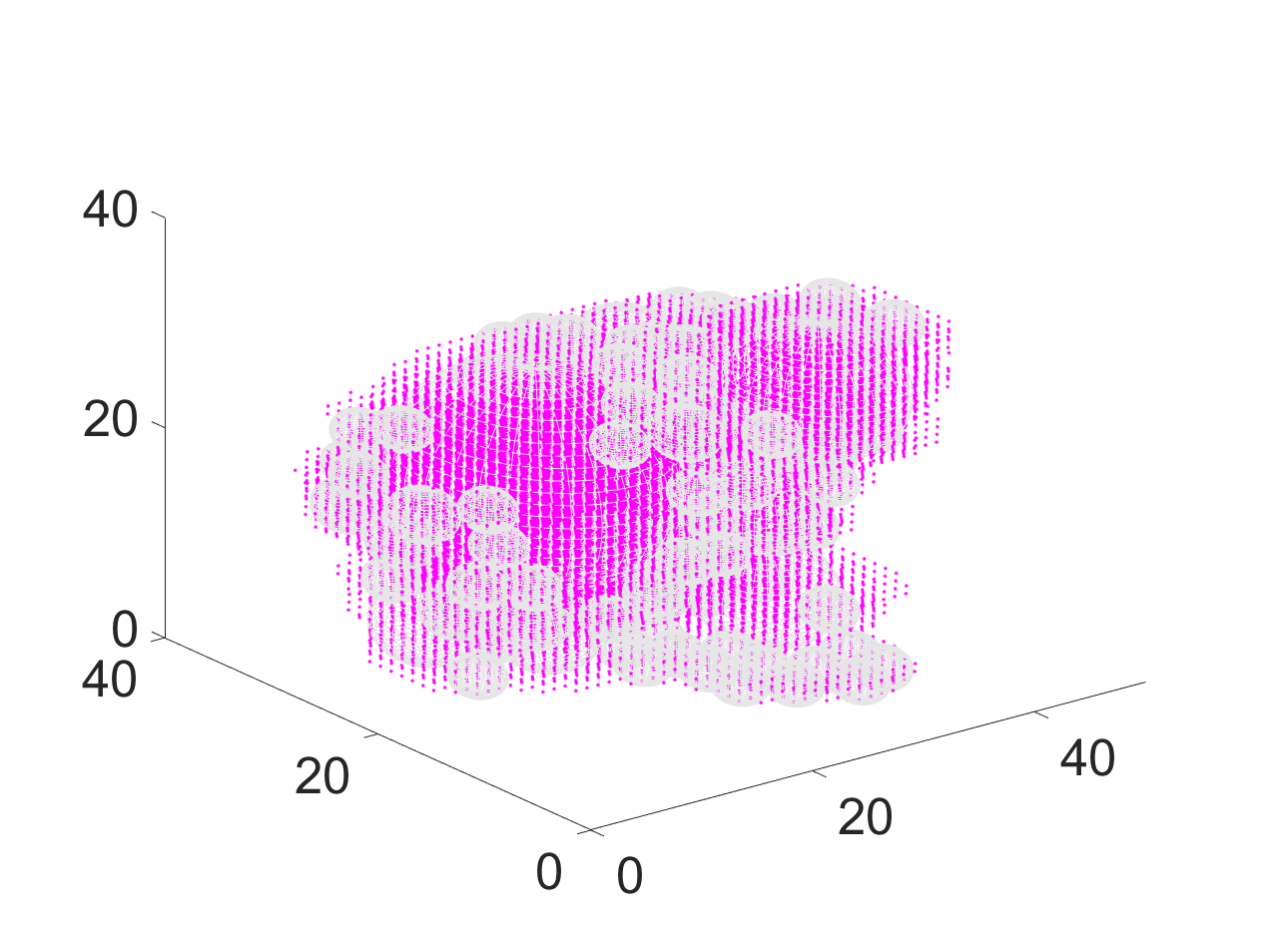
Sphere pack for case 2 with zero spacing (115 spheres)

Given the complexity of the input shape and the simplicity of the cylindrical beam shapes, the result in Figure 9 is impressive and most likely cannot be accomplished with optimization tools other than linear or quadratic programming. We then applied the same computations to two more cases. We will refer to these cases as Cases 2 and 3. Case 2 was a larger tumor of 19,951 cubic millimeters. Case 2 illustrates the importance of the sphere spacing parameter discussed above. With spacing set to zero, our algorithm is able to pack 115 spheres into the target (Figure 10). This would require substantial treatment time. We thus set the sphere-spacing (see section within Materials and Methods) to the value 3 mm and obtained a packing of 24 spheres. The CI and GI values obtained for this case are CI = 1.16 and GI = 2.67.

Case 3 was a mid-size tumor of 6,265 cubic millimeters. In this case, under a spacing of 1 mm, we obtained a packing with 19 spheres. The values CI and GI for case 3 are CI = 1.19 and GI = 2.71, respectively. Finally, to underline the importance of weight computation, we compared the weighted case to the non-weighted case; thus, use sphere packing alone, and simply assign even weights to all beams. The results obtained for this case are clearly inferior to the results obtained with linear or quadratic programming.

## Discussion

The values obtained for the CI and GI for the three cases are in agreement with CI and GI values achievable with state-of-the art technology [11]. However, a number of potential improvements have not yet been considered in our evaluation with only three cases. In addition, more detail on treatment delivery time is needed to design a full treatment planning algorithm. A factor influencing gradients is the size of the collimator. Using smaller collimators, it is often possible to produce steeper gradients. The possibility of placing smaller spheres in the vicinity of critical regions during sphere-packing (thereby sharpening gradients locally) has not yet been explored. Finally, it would also seem feasible to modify the output energy of the linear accelerator locally.

In Gamma Knife treatment protocols, spheres are allowed to intersect. Here, we do not allow intersections between spheres. In principle, this constraint could also be relaxed in our cases. However, as an important difference to the Gamma Knife, a robotic LINAC-based system allows for beam weighting. Quadratic programming is a tool from mathematical optimization. Based on mathematical principles, quadratic programming is guaranteed to find the global optimum of the user-defined objective function. We apply quadratic programming in such a way that dose gradients, conformity, or both are optimized in a least-square sense. Thus, the weighting step alone is globally optimal. Unfortunately, beam placement is still heuristic. Simple examples show that an exhaustive search for all possible beam placements is not possible due to a combinatoric explosion. However, as a practical advantage of quadratic programming (which addresses this problem), we find that quadratic programming returns a large number of beams receiving zero weights (typically more than 70% of the input beams receive weight zero, as noted). Quadratic programming can thus be used as an automatic (and globally optimal) beam selector. It is useful to begin the optimization process with a very large number of beams and refine this beam set iteratively. The interactive planning process relies on a number of input parameters, such as the spacing between spheres, shell distance, dose thresholds, and the number of beams, so that it would seem possible to search parts of this parameter space by semi-automatic methods, thereby further optimizing the treatment plan.

## Conclusions

We simulated dose distributions achievable with a new system for self-shielded radiosurgery. This system is currently under development. The results suggest that a larger database of cases should be analyzed with an automatized version of the proposed methods in order to obtain more detailed design recommendations for a treatment planning system for self-shielded radiosurgery. Then, a complete evaluation of this treatment planning system (once implemented) can be carried out in the future.
